# Psychometric properties of primary health care trust questionnaire

**DOI:** 10.1186/s12913-019-4340-6

**Published:** 2019-07-19

**Authors:** Homayoun Sadeghi-Bazargani, Mostafa farahbakhsh, Jafar Sadegh Tabrizi, Zahra Zare, Mohammad Saadati

**Affiliations:** 1 Evidence-Based Medical Research Center, Tabriz University of Medical Sciences, Tabriz, Iran; 2 Research Center of Psychiatry and behavioral Sciences, Tabriz University of Medical Sciences, Tabriz, Iran; 3 Tabriz Health Services Management Research Center, Tabriz University of Medical Sciences, Tabriz, Iran; 4 Department of Epidemiology, Faculty of Health, Tabriz University of Medical Sciences, Tabriz, Iran; 5 Road Traffic Injury Research Center, Tabriz University of Medical Sciences, Tabriz, Iran

**Keywords:** Public trust, Primary health care, Measurement tools, Validity, Reliability, Psychometrics

## Abstract

**Background:**

Trust has been introduced as the cornerstone of the public and health providers’ relation. Public trust in primary health care (PHC) is crucial and must be measured. The aim of this study was to develop and validate PHC trust measurement tool.

**Methods:**

This was a psychometric study to develop PHC trust measuring tool done in Tabriz, East-Azerbaijan with participation of 600 households in 2016. Item generation was done through literature review and experts opinions. The content validity, reliability and construct validity of the PHC trust tool were assessed using several statistical methods including modified Kappa, Kendall’s Tau and intra-class correlation coefficient (ICC) as well as exploratory factor analysis (EFA). Data were analyzed using STATA 14 statistical software package.

**Results:**

A 30-item questionnaire was developed. The Modified Kappa coefficient as an indicator of content validity assessment was 0.94. With respect to reliability assessment, a high internal consistency was observed with 0.98 Cronbach-Alpha score and the test–retest reliability for overall scale (assessed by ICC) was 0.94 (CI: 0.87–0.97). Exploratory factor analysis emerged 2 factors. Factor 1 consisted of 25 items accounting for 74.1% of the variance (eigenvalue = 22.47) followed by Factor 2 consisting of 5 items accounting for 19.2% of the variance (eigenvalue = 1.6).

**Conclusion:**

PHC trust measuring tool could be used as a valid and reliable tool by health systems in Iran and similar contexts to investigate how they are trustful from the public viewpoint.

**Electronic supplementary material:**

The online version of this article (10.1186/s12913-019-4340-6) contains supplementary material, which is available to authorized users.

## Background

Trust has become a popular word in all human relations [1] and within health systems, it was associated with quality of services and patient and physician relation [2, 3]. In the context of health, trust refers to the patients optimistic believes about care providers who provide the best act for him/her [4, 5]. Interpersonal and public trust are two forms of trust in health context [5]. Trust as an intrinsic value in health system, leads to more adherence of patients to treatment, better disclosure of self-reported and sensitive information by patients, care continuity and more self-efficacy [6–8]. Trust is a result of good performance of health care providers and patients positive experience and satisfaction [9]. Improving patients trust through improving their level of satisfaction is among the most important goals in health systems. Trust in health context could be defined considering personal or institutional. Patient personal trust on health providers such as physicians, nurses and etc., which is the result of interpersonal relations [2, 7, 10] and institutional trust in hospital and insurance system, which is the result of patient experience with the institution [8, 11, 12]. In this regard, trust in health context must be effectively measured using valid and reliable tools. Several previous studies have done on developing tools for measuring trust in health. Some measured the patients trust in physicians and other providers [13, 14], some in whole health system [8, 11] and some in insurance system [15]. These tools were reported to have good psychometric validity [16]. Primary Health Care (PHC) was an ignored scope in trust studies. Moreover, most of the studies on trust measuring tools developing were done in developed countries.

Primary health care in Iran is among the successful PHC systems in the world and it achievements have been encouraged by World Health Organization (WHO) [17, 18]. No doubt, PHC system success strongly relies on public participation and trust. Trust in PHC is different from other parts of health system, especially in low and middle income countries like Iran. Literature indicated that there was no valid tool for measuring trust in whole PHC. Regarding, this study aimed to develop and evaluate the reliability and validity of Trust in PHC questionnaire.

## Methods

This was part of a comprehensive study conducted to investigate the effectiveness of the Health Complex Model reform in Iranian primary health care in East-Azerbaijan province. The complete study and intervention protocol was published before [19, 20]. One of the most important variables to be investigated was trust in PHC system. As there was no valid tool for trust measuring, below process was done for developing and validating PHC trust tool.

### Context

Iranian health system consist of three level of referral including primary health care (PHC) centers at start point, secondary- level health centers and hospitals and specialized hospitals in tertiary level. PHC services are provided through health-houses, by trained person called behvarz who covers 1200 inhabitants, and rural health centers, by a physician and a team of health workers covering almost 7000 people, for rural population. Urban health centers provide similar services in urban areas, staffed with certified physicians and Community Health Workers, CHWs. Essential health services provided by Iran PHC are health education, access to healthy environment and drinking water, mother and child health, vaccination, communicable and non-communicable disease prevention, mental health and providing essential drugs [17, 21].

### Item generation and selection

Literature review through electronic databases (PubMed, ScienceDirect, Scopus and Web of Knowledge) was done. Trust, Health care, PHC, Public, tool and Item were the keywords used for literature review in published literature from 2000. After literature screening, a preliminary list of items related to trust in PHC was extracted, independently by two of the researchers (MF and MS). Then, the lists were merged. An expert panel session was held to review the extracted items. However, to address the issues not adequately covered by items, new items were generated by experts with relevant fields of medicine, psychology, health services management, Epidemiology, Health education and public health. Finally, a list of items was generated. The answers for each item of the questionnaire have five Likert-scaled choices as “very low = 0”, “low = 1”, “no idea = 2”, “high = 3” and “very high = 4”.

### Content validity

The content validity of the tool could be assessed through the experts’ viewpoint. Experts includes professionals who are content expert or have research or work experience in the topic [22]. Experts assess each item qualitatively in case of grammar, order of words, using correct and appropriate words and scoring. To assess the quantitative content validity by the experts, a questionnaire (Table 1) was developed. Experts were requested to rate the items using 4-point evaluation scales in case of item each necessity, relevance and clarity (Table 1).

**Table 1:** Content validity assessment form

Item	Necessity	Relevance	Clarity
	1(not useful)	1(not relevant)	1(not clear)
	2(not necessary)	2(item need some revision)	2(item need some revision)
	3(*useful but not essential*)	3(relevant but need minor revision)	3(clear but need minor revision)
	4(necessary)	4(completely relevant)	4(very clear)

Based on the experts scoring, Modified Kappa coefficient was computed. Multi-rater Kappa coefficient was introduced by literature, which adjust for chance agreement. Modified Kappa coefficient was calculated using the formula reported by Sim et al. 2005 [23].

$$ Kappa=\frac{P0- Pc}{1- Pc} $$

Where Po is the proportion of observed agreements and Pc is the proportion of agreements expected by chance.

Experts (*n* = 15) contained individuals with expertise in public health and working experience in PHC system more than 10 years (*n* = 7), Health Services Management specialists (*n* = 2), Epidemiologist (*n* = 2), Psychiatrist (*n* = 1), Medical Doctor (*n* = 2) and Health Education specialist (*n* = 1).

### Reliability

The reliability of the scale was measured using internal consistency and the test-retest method Cronbach alpha was used to measure the internal consistency for the total scale, and each subscale. For test-retest reliability of the scale, the Intra-class Correlation Coefficients (ICC) over a 15-day interval was calculated for total scale. In addition, Kendall’s Tau and Pearson Correlation Coefficients were reported for test-retest reliability. Tool feasibility was assessed through a pilot study including 30 households in Tabriz.

### Structural validity

Exploratory factor analysis using principal factor analysis with varimax rotation was conducted. Orthogonal rotation (varimax) was used assuming that various levels of public trust in PHC are not essentially considered to be correlated. In order to control the number of factors as well as through examination of the scree plot, minimum eigenvalue was set at 1. A uniqueness score below 0.7 was considered as the criterion for selecting items having adequate communality.

However, based on the six-component validity assessment framework provided by Messick (1995), validity evidences were reported in Additional file 1 [24].

### Sampling and data collection

Two-stage cluster sampling method through probability proportional to size (PPS) was used for sampling in Tabriz city. Study sample composed of 1,200 households (i.e., 60 clusters of 20 households). Tabriz population census forms, 2014, was used as sampling framework.

Based on the main data collection plan, presented in the protocol [19], households number 6–10 (600 households) were asked to response the PHC trust questionnaire. Head of the households or housewife were interviewed by a trained questioner. If he/ she was not able to respond, an educated member of the household of at least 15 years old was asked to respond. Each household was approached for data, 3 times. Household with a residence of less than 6 months in each area was excluded.

## Results

### Samples characteristics

Majority of the participants were female (55.26%) and married (83.49%). The mean age of the participants was 40.48 (± 14.97). Most of the participants (68.62%) have used social security insurance.

### Item generation and selection

A total of 42 items were generated through literature review. Researchers reviewed the preliminary items and some items were modified or merged. A preliminary list of 39 items were discussed by the experts. Four of items were eliminated, 4 were merged in 2 items based on the experts opinions. Moreover, some grammatical editions were made on the items.

### Content validity

The content validity of the Primary Health Care Trust was assessed based on both qualitative (comments from panel members) and quantitative methods. The Kappa coefficient was calculated as 0.94 based on the experts scoring.

### Reliability

The Cronbach-Alpha for overall scale, subscale 1 and subscale 2 was 0.98, 0.99 and 0.93, respectively. Test–retest reliability for overall scale (assessed by ICC) was 0.94 (CI: 0.87–0.97). Pearson correlations, Kendall’s tau-a and Kendall’s tau-b correlations were 0.89, 0.77 and 0.78, respectively for the scale.

### Structural validity

A model with two factors including 30 items emerged from principal factor analysis and subsequent scree test that accounted for 93.3% of the variance. The Kaiser–Meyer–Olkin (KMO) index was 0.98. The value of Bartlett’s test of sphericity was obtained 37104/808. Bartlett’s test of sphericity was significant (*P* < .001).

In the factor analysis, Factor 1 consisted of 25 statements accounting for 74.1% of the variance (eigenvalue = 22.47) which we labeled ‘*Main Factor*’. Factor 2 consisted of 5 statements accounting for 19.2% of the variance (eigenvalue = 1.6) which we labeled ‘*Specific or Optimal task* ’. Uniqueness of the all items was less than 0.7. An item- factor table from the rotated factor matrix is shown in Table 2. The final Questionnaire items are reported as Additional file 2.

**Table 2:** factor loadings of 30 items

Items	^a^Factor1	^b^Factor2	Uniqueness
	+	+	
1	0.9123	0.3795	0.0237
2	0.9102	0.3926	0.0174
3	0.6782		0.4604
4	0.4023	0.9041	0.0208
5	0.6608		0.4848
6	0.9120	0.3902	0.0160
7	0.9103	0.3910	0.0185
8	0.6686		0.4737
9	0.6687		0.4729
10	0.6624		0.4764
11	0.9069	0.4030	0.0151
12	0.9045	0.4031	0.0194
13	0.9046	0.4047	0.0179
14	0.9092	0.3944	0.0177
15	0.6517		0.4872
16	0.6651		0.4703
17	0.6607		0.4828
18	0.9090	0.3933	0.0190
19	0.9009	0.4070	0.0226
20	0.8953	0.4014	0.0374
21	0.6456		0.4980
22	0.8863	0.4048	0.0506
23	0.3227	0.7563	0.3240
24	0.6486		0.4950
25	0.3240	0.7517	0.3300
26	0.3925	0.9078	0.0218
27	0.8819	0.4042	0.0588
28	0.9000	0.3967	0.0327
29	0.8999	0.3977	0.0321
30	0.9064	0.3713	0.0405

Blanks represent absolute loading, 0.3

Uniqueness = 1-communality

The values for the items included in each of the factors are shown in bold underneath the heading for that factor

^a^Main Factor

^b^Specific or Optimal task

## Discussion

The result showed strong internal consistency and reliability and also good validity of Iranian PHC trust questionnaire. A model with two factors with 30 items emerged from principal factor analysis and subsequent scree test that accounted for 93.3% of the variance. The uniqueness indexes of all items were less than 0.5 and this suggests a good relevance of the items.

Literature have reported the consistency of available trust measuring tools, ranging from 0.74 to 0.93, as a high level of consistency [16]. Our results indicated higher Cronbach-Alpha for overall scale as 0.98. Moreover, it was calculated for factor 1 and factor 2, about 0.99 and 0.93, respectively. These results showed the high consistency of the Iranian PHC trust questionnaire.

Hall et al. (2002) had developed a tool to measure trust in primary care physicians. The tool was focused on physicians in 10-items and one factor. Its Cronbach-Alpha index was 0.93 [10]. We have developed a tool measuring patients trust in whole primary care system through 30 items in 2 factors with α = 0.98. Moreover, this was higher than the consistency of tool developed by Straten et al. (2002) to measure trust in whole health system [25].

Previous studies developing tools for measuring trust in health care were not worked on measuring trust in whole primary care [16]. Iranian PHC trust tool, with a strong validity and reliability, provide a tool for quantitative analysis of public trust in PHC system in Iran.

PHC as the first level of health services delivery and covering a wide range of population, needs to be more trustful than other levels. This will improve the PHC services effective coverage and ultimately decreases the health system costs [26]. To the best of our knowledge, this tool was the first measuring tool for trust in PHC. The tool could be used by other countries to evaluate their population trust in PHC. Moreover, measuring PHC trust in other countries provides data for international comparisons and also investigating determinants of PHC trust across various cultures.

## Conclusions

The initial development and validation of PHC trust measuring tool was done in the study. Result showed that the tool has a strong consistency and good validity and reliability to be used. The tool could be used to measure public trust in PHC system in Iran and similar countries.

### Limitations

We did not used public opinions in the tool development and judgment process. Moreover, most of the similar studies done in a small community and their use in various settings provide the tool generalizability and improvement of the tool external validity.

## Additional files

Additional file 1: Validity evidences. PHC Trust questionnaire validity evidences based on six-component validity assessment framework provided by Messick (1995). The table provide Trust questionnaire validity evidences based on six-component validity assessment framework provided by Messick (1995). (DOCX 14 kb)

Additional file 2: PHC-TrustQ. Final PHC Trust Questionnaire Items. The table provide items which were finalized as PHC Trust Questionnaire. (DOCX 15 kb)

## Data Availability

The datasets used and/or analyzed during the current study are available from the corresponding author on reasonable request.

## Abbreviations

CHW: Community Health Worker
ICC: Intra-class Correlation Coefficients
KMO: Kaiser–Meyer–Olkin
PHC: Primary Health Care
PPS: Probability Proportional to Size
WHO: World Health Organization
