# Supplementary material for: Psychometric properties of primary health care trust questionnaire
Source: BMC Health Serv Res. 2019 Jul 19;19:502. doi: 10.1186/s12913-019-4340-6 (PMC6642567; doi:10.1186/s12913-019-4340-6)
Supplement: Supplementary file 1 — Validity evidences. PHC Trust questionnaire validity evidences based on six-component validity assessment framework provided by Messick (1995). The table provide Trust questionnaire validity evidences based on six-component validity assessment framework provided by Messick (1995). (DOCX 14 kb) [file 12913_2019_4340_MOESM1_ESM.docx]

| Type of evidence | Aspect of Validity | Validity Evidence |
| --- | --- | --- |
| Data based evidence | Structural | ■ Demonstrating internal relationships among items (structural fidelity) or subsets of items consistent with the underlying theory (factor analysis, item difficulty hierarchies that are consistent with the construct map)  ■ Demonstrating that conditional difficulties of items are equal or consistent with known influences on test performance (differential item functioning analysis)  ■ Utilizing a measurement model that combines (weights) information across observations (scales) and takes into account (controls for) undesirable influences on the scores in a manner that is cons |
|  | Generalizability | ■ Demonstrating reliability of measures from the instrument across a variety of contexts  ■ Internal consistency  ■ Test-retest  □ Inter-rater  □ Alternate form  □ Application of meta-analytic procedures to validity coefficients across a variety of measurement contexts and samples (validity generalization) |
|  | External | □ Demonstrating that predicted group differences are realized empirically.  □ Experimentation: Any theory-based comparison that yields outcomes consistent with theory.  □ Comparison of Groups: Differences between groups that theory predicts will be different on the underlying construct  □ Changes Over Time: Individuals are typically expected to change over time as a result of maturation. Observed differences such as these can also be used as evidence for construct validity. |
|  | Consequential | ■ Detecting positive or negative impact on individuals (bias) (e.g., identifying substantively explainable DIF versus sources of item or test bias, determining whether the labels generated in test use result in stigmatization of some groups, expert judgment about the suitability of test content for making decisions about individuals and groups) |

Additional file 1

PHC Trust questionnaire validity evidences based on six-component validity assessment framework provided by Messick (1995)

| Type of evidence | Aspect of Validity | Validity Evidence |
| --- | --- | --- |
| Test based evidence | Content | ■ Documentation of purpose & uses of the instrument  ■ Documentation of use of domain analysis  ■ Development of test blueprint & item templates  ■ Documentation of test development process  ■ Expert Review (Readability, Clarity, Clarity of instructions, Fairness, Sensitivity) |
|  | Substantive | ■ Development of operationalized definition & theoretical framework of the construct  (including internal, external, & processing models)  ■ Documentation of use of expertise  □ Verifying of use of the proposed processes by respondents (think aloud) |
|  | Structural | ■ Development of internal model  □ Rationale for adoption of reference framework (criterion versus normative)  ■ Rationale for developing response format (rating scale and/or distracters)  ■ Rationale for adoption of measurement model |
|  | Generalizability | ■ Specification of target population  ■ Selection of norming population |
|  | External | □ Demonstrating that the construct theory is sufficiently developed to support investigations of external aspect evidence |
|  | Consequential | □ Detecting positive or negative impact on systems (systemic validity) |
