# Supplementary material for: Psychometric properties of primary health care trust questionnaire
Source: BMC Health Serv Res. 2019 Jul 19;19:502. doi: 10.1186/s12913-019-4340-6 (PMC6642567; doi:10.1186/s12913-019-4340-6)
Supplement: Supplementary file 2 — PHC-TrustQ. Final PHC Trust Questionnaire Items. The table provide items which were finalized as PHC Trust Questionnaire. (DOCX 15 kb) [file 12913_2019_4340_MOESM2_ESM.docx]

**Final PHC Trust Questionnaire Items**

| N | Item | Very high | High | NA | low | Very low |
| --- | --- | --- | --- | --- | --- | --- |
| 1 | I'm confident doctors take the patient's condition seriously. |  |  |  |  |  |
| 2 | I'm confident Health workers take the patient's condition seriously. |  |  |  |  |  |
| 3 | I'm confident that patients can easily access doctors in their health care units. |  |  |  |  |  |
| 4 | I'm confident that patients can easily access health workers in their health care units. |  |  |  |  |  |
| 5 | I'm confident that doctors give enough time to listen to the questions and concerns of their clients. |  |  |  |  |  |
| 6 | I'm confident that Health workers give enough time to listen to the questions and concerns of their clients. |  |  |  |  |  |
| 7 | I am confident that in the health centers, doctors spend enough time to address the client's problem. |  |  |  |  |  |
| 8 | I'm confident that doctors respect the clients' rights. |  |  |  |  |  |
| 9 | I'm confident that Health workers respect the clients' rights. |  |  |  |  |  |
| 10 | I am confident that the physician's behavior is respectful and satisfactory. |  |  |  |  |  |
| 11 | I am confident that the health workers behavior is respectful and satisfactory. |  |  |  |  |  |
| 12 | I am confident that doctors and health workers are sympathetic to the client'. |  |  |  |  |  |
| 13 | I am sure that doctors and health workers respond the clients' needs. |  |  |  |  |  |
| 14 | I am confident that I will receive the required services when I visit the health center, without delay. |  |  |  |  |  |
| 15 | I'm confident the health services that are being provided at health centers are low risk and will not harm me. |  |  |  |  |  |
| 16 | I am confident that the government has an acceptable monitoring on services quality, provided in health care facilities. |  |  |  |  |  |
| 17 | I'm confident that doctors have enough knowledge and skills to meet the client's needs. |  |  |  |  |  |
| 18 | I'm confident that health workers have enough knowledge and skills to meet the client's needs. |  |  |  |  |  |
| 19 | I'm confident the health and medical supplies, needed by the clients, are provided at health centers. |  |  |  |  |  |
| 20 | I'm confident that my secrets will not be seamless outside the health center. |  |  |  |  |  |
| 21 | Due to the health center supervision, I am confident in the health of the food I provide. |  |  |  |  |  |
| 22 | The Health centers offers the best vaccine services. |  |  |  |  |  |
| 23 | I'm confident the healthcare centers provide the best care for pregnancy. |  |  |  |  |  |
| 24 | Due to the health center supervision, I am confident in the health of my drinking water. |  |  |  |  |  |
| 25 | I'm confident that the health center will provide the most useful information on family counseling. |  |  |  |  |  |
| 26 | I am confident that health centers play a crucial role in critical situations and the prevalence of serious illnesses. |  |  |  |  |  |
| 27 | Due to the supervision of the health unit, I am confident of the schools health and safety. |  |  |  |  |  |
| 28 | In case of need to health consulting, the health center will provide the most useful and effective information. |  |  |  |  |  |
| 29 | I am fully confident in the health center physician, as I share all of my medical needs with him. |  |  |  |  |  |
| 30 | Suppose you went to get the health unit service after receiving the service, the next time you visit have been announced. If you do not see at the designated time, how confident are you that the doctor or health worker will follow up? |  |  |  |  |  |
